# Creating ophthalmology experiences in undergraduate medical education: pilot of a cased-based learning ophthalmology tool

**DOI:** 10.1186/s12909-023-04514-8

**Published:** 2023-08-09

**Authors:** Jessica H. Tran, Emma Loebel, Mark Edouard, Thomas Quehl, Erin Walsh, Robin Ginsburg, Tameisha Frempong, Douglas Fredrick, Laura K. Stein, Michael G. Fara, Samira S. Farouk, Nisha Chadha

**Affiliations:** 1 Department of Ophthalmology, Icahn School of Medicine at Mount Sinai/New York Eye & Ear Infirmary, Eye and Vision Research Institute, New York, USA; 2 Department of Medical Education, Icahn School of Medicine at Mount Sinai, New York, USA; 3 Department of Neurology, Icahn School of Medicine at Mount Sinai, New York, USA; 4 Department of General Preventative Medicine, Icahn School of Medicine at Mount Sinai, New York, USA; 5 Department of Pediatrics, Icahn School of Medicine at Mount Sinai, New York, USA; 6 Department of Ophthalmology & Visual Science, Yale School of Medicine, New Haven, USA; 7 Kaiser Permanente Medical Group South San Francisco, San Francisco, USA; 8 Barbara T. Murphy Division of Nephrology, Department of Medicine, Icahn School of Medicine at Mount Sinai, New York, USA

**Keywords:** Medical education, Virtual learning, Trainees, Clerkship, Problem-based learning, Clinician educators

## Abstract

**Purpose:**

To evaluate medical student perceptions of a novel ophthalmology resource delivered through facilitated workshops in the core clerkship curriculum.

**Methods:**

We created www.2020sim.com, a free case-based learning (CBL) ophthalmology tool, adapted from NephSIM (www.nephsim.com). The tool was first piloted with the internal medicine (IM) residents. After confirming a need, we focused on undergraduate medical education (UME) by expanding the 20/20 SIM content and partnering with the neurology (pilot academic year [AY] 2020-2021) and pediatric clerkships (pilot AY 2021-2022) to deliver a facilitated one-hour ophthalmology workshop within each clerkship’s didactic curriculum. We evaluated the tool using pre- and post-surveys and knowledge assessments.

**Results:**

Of 80 IM residents, 33 (41.3%) completed the needs assessment. Of the 25 residents who attended the workshop, 23 (92.0%) completed the exit survey. IM residents reported discomfort in several ophthalmology domains (9 of 14 rated mean score < 3.0), confirming a need. Most (*n* = 21/23, 91.3%) rated the tool as good/excellent. Of 145 neurology clerkship students, 125 (86.2%) and at least 88 (60.7%) students completed the pre- and post-test/exit surveys, respectively. On average, participants highly rated the tool, perceiving 20/20 SIM to be relevant to their education [4.1 (0.8)]. Mean pre- to post-test knowledge scores increased from 7.5 to 8.5/10.0 points (*p* < 0.001). Of the 136 pediatric clerkship students, 67 (49.3%) and 51 (37.5%) completed the pre- and post-surveys, respectively. Respondents perceived increased comfort with ophthalmology topics after the facilitated workshop [3.8 (0.8)]. Mean pre- to post-test knowledge scores trended from 1.8 to 2.0/5.0 points (*p* = 0.30). Collectively, 20/139 (14.4%) of exit survey respondents visited www.2020sim.com within 1 month after the workshop.

**Conclusion:**

After identifying areas of greatest need with residents, we partnered with core clerkships to deliver cross-disciplinary ophthalmology content in UME. We found high engagement with 20/20 SIM, with trends toward increased knowledge.

## Introduction

Frontline providers are often the first to evaluate eye complaints [1–4]. However, they report low confidence in ophthalmic evaluation, which may hinder their ability to recognize non-vision threatening versus vision-threatening presentations for appropriate triage and referral [4–6].

Increasing curricular time for ophthalmology education is a strategy to address this gap [7]. The International Council of Ophthalmology (ICO) Task Force recommends 40–60 h of ophthalmic instruction in undergraduate medical education (UME) [8]. However, a cross-sectional survey in the United Kingdom suggested that medical schools did not meet that standard [9]. In the United States (U.S.), the Liaison Committee on Medical Education (LCME) and Accreditation Council for Graduate Medical Education (ACGME) do not provide guidelines for an ophthalmology curriculum. Accordingly, ophthalmology education has declined, especially during the clinical phase in UME [10, 11]. While > 90% of medical schools report preclinical exposure to ophthalmology, only 16–18% require students to complete an ophthalmology rotation [10, 11]. In primary care residency programs, program directors reported that less than 50% of their incoming residents met the ophthalmic core competencies established by the Association of University Professors of Ophthalmology (AUPO) [12].

Elective courses have been increasingly utilized to further medical students’ exposure to ophthalmology [10, 13–16]. In particular, the development of virtual ophthalmology rotations during the coronavirus 2019 (COVID-19) pandemic represented a crucial step forward for ophthalmic education, with several studies showing increased confidence and interest in the field among participants [14–16]. However, as electives are voluntary in nature, the provision of comprehensive basic training to the broadest audience of physicians is limited [7]. Furthermore, the intermittent nature of such sessions and lack of repetition make knowledge retention and application to future clinical encounters challenging [17]. To address this need, we sought to increase ophthalmology exposure by 1) partnering with core clerkships to identify didactic opportunities within the required UME curriculum and 2) introducing a novel, free, web-based ophthalmology learning tool through a facilitated workshop, which could then be revisited independently to reinforce learning. This single-site study evaluates the use of the educational tool, “20/20 SIM,” (www.2020sim.com), in a three-phase pilot with the: 1) internal medicine (IM) residency program, 2) neurology clerkship, and 3) pediatric clerkship, targeting three medical specialties likely to encounter patients with ocular and visual complaints.

## Methods

**Fig. 1:**
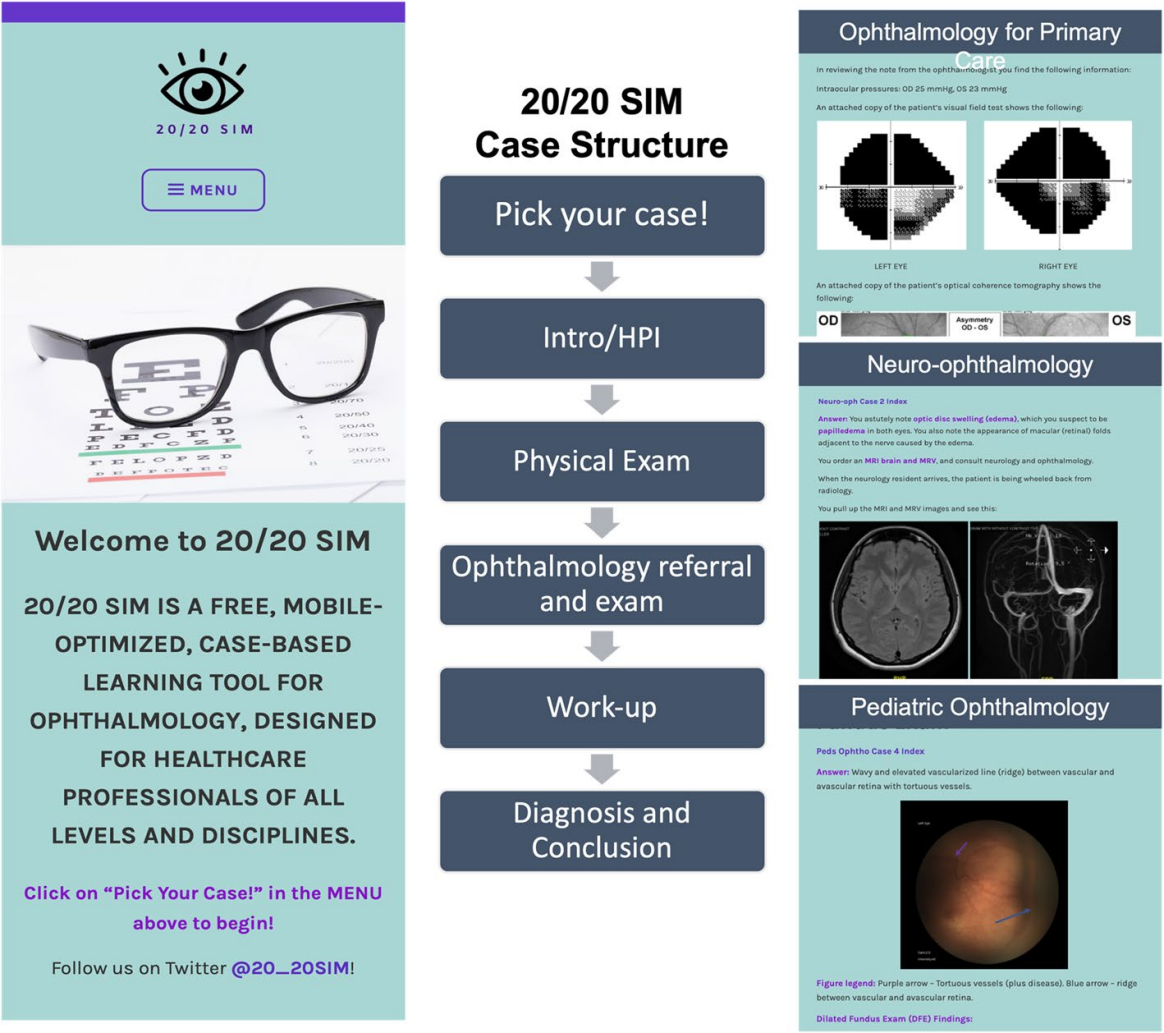
Snapshot of the 20/20 SIM website interface and structure (www.2020sim.com)

**Fig. 2:**
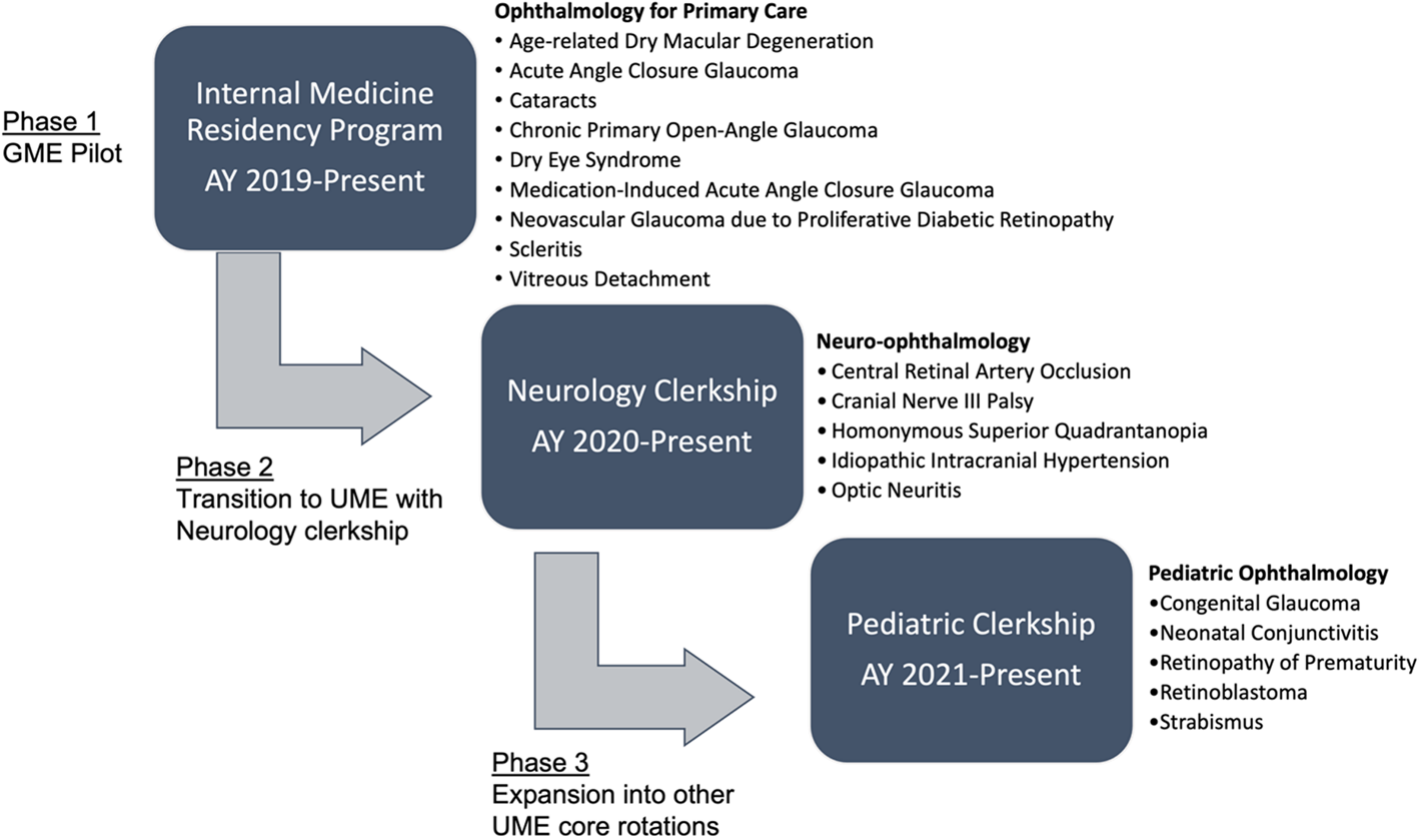
Study design and integration of the 20/20 SIM tool in graduate medical education (GME) and undergraduate medical education (UME) at the Icahn School of Medicine at Mount Sinai. Each series of the 20/20 SIM tool was introduced into the required didactic curriculum through a facilitated workshop where a faculty investigator led a guided walkthrough and discussion of some of the 20/20 SIM cases. Abbreviations: AY, academic year; GME, graduate medical education; UME, undergraduate medical education

20/20 SIM is a free, web-based educational tool that uses interactive cases with multimedia and self-assessment questions to teach ophthalmology to learners from all disciplines (Fig. 1). It is an adaptation of NephSIM (www.nephsim.com), a free open access medical education (FOAMed) resource for nephrology [18]. 20/20 SIM was the first collaborator in the “SIM series,” which has since expanded to other specialties, including neurology (NeuroSIM, www.neurologysim.wordpress.com), gastroenterology (GI SIM, www.gi-sim.com), and rheumatology (RheumSIM, www.rheumsim.com) [18]. The SIM series follow a case-based learning (CBL) style, in which learners work through a real-world scenario to facilitate clinical reasoning and knowledge acquisition in diagnosis and management [7, 19]. For 20/20 SIM cases, users are presented with information in successive order, starting with the history of present illness (HPI), physical examination, diagnosis, and management. Uniquely, each case also reviews the ophthalmic evaluation and work-up so that learners may gain understanding of the management following referral. To facilitate structured learning, users can answer multiple choice questions before advancing to the next portion of the case. Questions are tailored toward clinical reasoning development and include the formulation of differential diagnoses, data interpretation, and clinical decision making (Fig. 1). All case topics (Fig. 2) were developed using the learning objectives from the AUPO, American Academy of Neurology (AAN), and Council on Medical Student Education in Pediatrics (COMSEP), as well as consensus among our multispecialty investigator group including ophthalmologists, neurologists, and internists (LS, MF, NC, DF, EW, ME) [20–22]. The 20/20 SIM website was developed using WordPress, a web publishing software (WordPress Foundation, San Francisco, CA).

### Development of 20/20 SIM cases and its integration into the core curriculum

Figure 2 summarizes the three-phase study design. Briefly, we hosted facilitated workshops using the 20/20 SIM tool for trainees, which was led by at least one faculty study investigator. Each workshop began with an anonymous pre-test. Participants were not asked to review the online cases prior to the workshop. The facilitator then introduced and led a guided 20/20 SIM case walkthrough and interactive real-time discussion as one large group (~ 15 trainees/session). The discussion included a review of the case learning objectives and commentary on the self-assessment questions embedded within the case. Each facilitated case took at least 20–25 min to complete. An anonymous voluntary post-test was completed 2–4 weeks later. Pre- and post-test questions were different from the case self-assessment questions. Additional details are described below.

### Internal medicine residency program

**Fig. 3:**
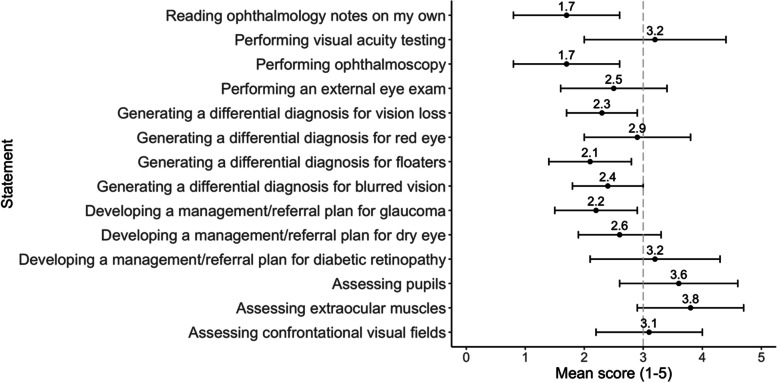
Internal medicine resident perceptions (*N* = 23) of comfort level with ophthalmology work-up, diagnosis, and management based on a mean score where 1=very uncomfortable and 5=very comfortable. The horizontal bars reflect one standard deviation in each direction. The vertical dashed line indicates a mean score of 3 (neutral).

For the initial pilot (academic year [AY] 2019–2020), we developed nine "ophthalmology for primary care” cases and partnered with the IM residency program to deliver a workshop using the cases as part of the didactic curriculum for postgraduate year (PGY)-2 and PGY-3 residents (Fig. 2). The goal of the series was to develop primary care-oriented cases that were accessible to learners with minimal ophthalmic knowledge. As such, topics included “bread and butter” complaints such as mature cataracts to clinical scenarios that challenged learners to consider the sequelae of chronic eye problems such as neovascular glaucoma in the setting of proliferative diabetic retinopathy. The one-hour in-person workshop, delivered on three occasions, covered a facilitated case review selected by the learners and a basic ophthalmology physical exam skills session. The positive reception, along with confirmation of educational needs during the initial pilot with the residents (Fig. 3), led to the development of workshops within UME.

### Neurology clerkship

As ocular complaints may present in the setting of neurologic symptoms and require initial work-up by the neurologist, we partnered with the neurology core clerkship to expand 20/20 SIM to include neuro-ophthalmology cases. We developed five cases, aligned with the goals of the clerkship, and delivered a 1-h facilitated workshop (12 total) for all third-year medical students during their neurology rotation (Fig. 2). For the pilot year (AY 2020–2021), the neuro-ophthalmology workshops were adapted to a virtual video conference format due to the COVID-19 pandemic. For these workshops, cases were chosen at random with 1–2 cases covered per session.

### Pediatric clerkship

Following the UME pilot with the neurology rotation, we formed a partnership with the pediatric core clerkship, as pediatricians often screen children for visual complaints. For this phase, we developed five pediatric ophthalmology cases and hosted a 45-min facilitated workshop (8 total) for all third-year medical students during their pediatric clerkship (Fig. 2). In the pilot year (AY 2021–2022), the pediatric ophthalmology workshop focused on retinoblastoma or strabismus. The workshops were implemented in a hybrid format (video conference and in-person) to allow students at different clinical sites to attend.

### Evaluation of workshop

As the workshop was ≤ 1 h in length, the primary outcome was participant satisfaction. All participants were encouraged to complete anonymous pre- and post-surveys that were administered through Google Forms (Google, San Francisco, CA) or Research Electronic Data Capture (REDCap) (Vanderbilt University, Nashville, TN). In phase 1 of the 20/20 SIM pilot, the pre-survey was an 18-item needs assessment querying IM residents on their comfort with common ophthalmic presentations and exam skills. Items on the exit survey pertained to the quality and satisfaction with the 20/20 SIM tool in workshop format. For the UME pilots (phase 2 and phase 3), we measured participant satisfaction of the CBL tool through an exit survey and secondarily, assessed knowledge through pre- and post-tests. The assessments were related to diagnosis and management of all neuro-ophthalmology (10 questions – 2 questions/case) or pediatric ophthalmology conditions covered on 20/20 SIM (5 questions – 1 question/case) (see Fig. 2 for case topics). Therefore, the quiz questions included topics that were not taught during the workshop, as it was not feasible to review all cases in ≤ 1 h. All exit survey and post-test data were collected 2–4 weeks following the workshop. As no identifiers were collected, participants were able to complete the post-survey without pre-survey completion. No demographics were collected in the survey instruments.

For statistical analyses, Likert scale survey data are reported as mean scores (1–5) and standard deviation (SD). Open-ended responses were analyzed for recurring themes (JT, NC) [23, 24]. For the knowledge assessment, we evaluated pre- and post-test scores using unpaired Wilcoxon signed rank tests. A p-value of < 0.05 was considered statistically significant. All analyses were conducted in R Version 4.2.1 (R Foundation for Statistical Computing, Vienna, Austria). Reported data is from the first pilot year of each collaboration. The Institutional Review Board (IRB) at Icahn School of Medicine at Mount Sinai determined each study phase to be exempt (STUDY-20–00088, STUDY-21–01398, STUDY-21–00263) and waived the need for informed consent. All methods were carried out in accordance with relevant guidelines.

## Results

### Internal medicine residents

Of 80 residents, 33 (41.3%) completed the needs assessment and 25 (31.3%) participated in the workshop. Not all residents could attend the workshops due to clinical responsibilities or off-site rotations. Twenty-three (92.0%) of 25 workshop participants completed the exit survey. In the needs assessment, 19/33 (57.6%) of respondents reported prior eye education in the primary care setting. Of the 19 with prior ophthalmic training, 9 (47.4%) and 5 (26.3%) participants reported education during medical school and residency (i.e., intern ophthalmoscopy training), respectively. Only 3/19 (15.8%) stated they received training in both medical school and residency. Figure 3 summarizes participant perceptions of their comfort level with ophthalmology, with 9 out of 14 domains rated a mean score < 3.0/5.0. Higher scoring domains included comfort with basic eye exam skills (i.e., visual acuity, extraocular muscles, and pupils, mean scores all ≥ 3.1) and developing a management/referral plan for diabetic retinopathy [3.2 (1.1)]. Lower scoring domains included comfort with performing ophthalmoscopy [1.7 (0.9)], reading ophthalmology notes [1.7 (0.9)], developing a management/referral plan for glaucoma [2.2 (0.7)], and developing a differential diagnosis for common eye complaints (i.e., vision loss, red eye, and floaters, mean scores all ≤ 2.9). The greatest barriers to performing the eye exam were perceived discomfort with the exam (*n* = 32/33, 97.0%) and limited time (*n* = 25/33, 75.8%). When residents were asked what they enjoyed the most about the workshop, 9 (39.1%) of 23 exit survey participants reported the CBL tool and 4 (17.4%) cited both the CBL and physical exam practice components of the workshop. Some (*n* = 6, 26.1%) respondents included comments on the “interactive” nature of the workshop. Regarding case difficulty level, 14 (60.9%) residents felt the cases were “just right” with a mean rating of 3.4 (0.6). Meanwhile, 8 (34.8%) and 1 (4.3%) stated the cases were challenging or too challenging, respectively.

### Neurology clerkship students

Of 145 neurology clerkship students who attended the mandatory neuro-ophthalmology workshop, 125 (86.2%), 102 (70.3%), and 88 (60.7%) students completed the pre-test, post-test, and exit survey, respectively. Students rated the CBL workshop favorably (Table 1). Respondents found the workshop to be relevant to the core clerkship [4.1 (0.8)] and preferred the CBL workshop format to traditional didactics [3.9 (0.8)]. Participants perceived their knowledge to increase following the brief workshop [3.8 (0.8)], which was confirmed with their mean pre- and post-assessment scores [pre: 7.5 (2.2), post: 8.5 (1.6), *p* < 0.001]. Significant score gains were on exam items related to central retinal artery occlusion and optic neuritis. Students felt that the difficulty level of the the pre- and post-knowledge assessment was “just right” with a mean score of 3.0 (0.5).

**Table 1:** Student perceptions of the CBL tool from the pilot year where 1=strongly disagree and 5=strongly agree

Statement (1–5 Likert scale)	Neurology Rotation (*N* = 88) AY 2020–2021	Pediatric Rotation (*N* = 51) AY 2021–2022
	Mean (SD)	
I would recommend the tool to others	3.8 (0.9)	3.6 (0.9)
The CBL workshop was relevant to the core clerkship	4.1 (0.8)	4.1 (0.8)
I enjoyed the CBL workshop	3.7 (1.1)	4.1 (0.9)
I preferred the CBL workshop to traditional didactics	3.9 (0.8)	3.7 (1.0)
I felt that my knowledge^1^ or comfort^2^ in ophthalmology increased after the workshop	3.8 (0.8)^1^	3.9 (0.9)^2^

*AY* academic year, *CBL* case-based learning, *SD* standard deviation

^1^Students in the neurology clerkship were asked about perceived increased knowledge

^2^Students in the pediatric clerkship were asked about perceived increased comfort level

### Pediatric clerkship students

Of 136 students who attended the required pediatric ophthalmology workshop, 67 (49.3%) and 51 (37.5%) completed the pre-test and post-test/exit surveys, respectively. Like the neurology clerkship students, the students on the pediatric rotation found the workshop to be relevant to the goals of the clerkship [4.1 (0.8)] and preferred this method of learning to traditional didactics [3.7 (1.0)] (Table 1). Students perceived increased comfort with ophthalmology topics after participating in the workshop [3.9 (0.9)]. There was a nominal trend towards increased mean knowledge with the pediatric ophthalmology tool [pre: 1.8 (1.0), post: 2.0 (1.0), *p* = 0.30]. The pediatric clerkship students found the knowledge assessment to be significantly more difficult than the neurology clerkship students [3.5 (0.7) vs. 3.0 (0.5), respectively, *p* < 0.001].

### Overall UME perceptions

**Fig. 4:**
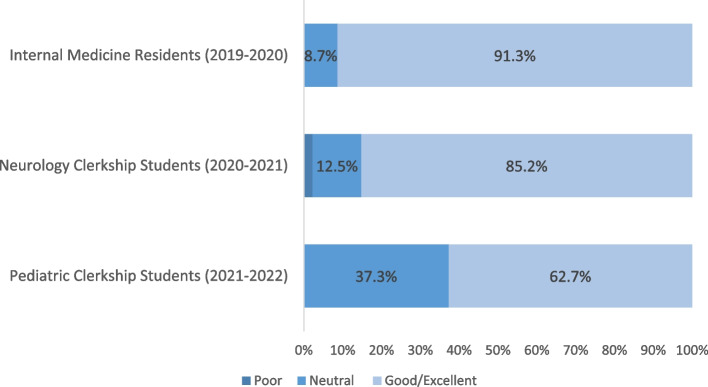
Resident (*N*=23) and clerkship student (neurology: *N*=88, pediatrics: *N*=51) perceptions of the quality of the 20/20 SIM tool

Similar to the perceptions from the IM residents, most surveyed students on the neurology (*n* = 75/88, 85.2%) and pediatric rotation (*n* = 32/51, 62.7%) rated the quality of 20/20 SIM as good or excellent (Fig. 4). Students on the neurology and pediatric clerkship reported that they would like to see the development of a similar tool for other specialties (*n* = 81/88, 92.0% and *n* = 44/51, 86.3%, respectively). Collectively, 14.4% (*n* = 20/139 exit survey respondents) reported visiting the 20/20 SIM website when surveyed 2–4 weeks after the workshop.

## Discussion

Over the past two decades, the number of ophthalmic curricular hours in UME has declined, with most instruction limited to the preclinical phase [10, 11]. While visual complaints may require initial evaluation and management by frontline primary care providers [2, 4, 25], many physicians, including those in our study, report low confidence in ophthalmology [4, 5]. This study introduced 20/20 SIM (www.2020sim.com), a free online CBL ophthalmology tool that is part of the broader “SIM series,” [18] to help address this educational need. While there are many FOAMed ophthalmology resources are available, those resources tend to target a specific audience (i.e., medical students vs. ophthalmology residents) with a user format that primarily supports self-guided learning. Our novel tool hopes to engage learners from all training levels and complement the existing content, using a sequential CBL format that is structured for both independent and easily adaptable group learning. We show how the well-rated tool may be integrated within required clinical experiences using facilitated workshops to incorporate additional ophthalmology education in UME. This study also suggests the accessibility of the free resource to further self-directed ophthalmic education.

In the pilot with the IM residents, most respondents received prior ophthalmology education. However, many reported discomfort with ophthalmology, particularly with generating a differential diagnosis for common visual complaints, performing fundoscopy, and reading ophthalmology notes. Aligned with previous reports [4–6], our study corroborated a need to increase ophthalmic education so future gatekeepers and relevant specialists can comfortably manage and triage ocular presentations. Of note, residents in this study positively rated the 20/20 SIM tool and perceived the cases to be appropriate in difficulty level, suggesting that the cases are well-suited for trainees in primary care fields. Taken together, these results suggested both a need and interest to adapt the tool to engage trainees earlier in their medical education.

Based on our findings with the IM residents, the study focused on the delivery of the 20/20 SIM tool to the broadest audience of medical trainees. As few medical schools require ophthalmology rotations, we targeted the clinical phase, specifically core clerkships, to reinforce and build upon ophthalmology concepts introduced in the preclinical stage. Lippa et. al demonstrated the potential benefits of increased ophthalmology education in the clerkship phase, with improvements in ophthalmic physical exam skills after students underwent a clinical refresher [17]. Uniquely, we focused our pilot on adding cross-disciplinary ophthalmology education to the core curriculum using a brief (45-min to 1-h) CBL facilitated workshop. Within ophthalmology education, this style of “flipped classroom” learning appears successful in enhancing participant engagement and academic outcomes [26–28]. In our study, we found that all three cohorts, despite receiving the workshop in different modalities (in-person, virtual, and hybrid format), were satisfied with the CBL tool. Moreover, consistent with previous literature, the students preferred the CBL workshop to traditional didactics, underscoring how active learning has become increasingly favored [19, 26–29]. The students also perceived the workshop to be relevant to their clerkship, with some participants visiting the 20/20 SIM website shortly after the session. In all, these results support the added educational value of integrating ophthalmology education in core rotations through 20/20 SIM and its potential utility as a future resource for learners to revisit content independently.

In both clerkships, we found that the brief workshop was associated with trends towards increased knowledge. While we only detected a significant knowledge increase from the neuro-ophthalmology workshops, we believe the null findings in the pediatric ophthalmology pilot were due to several factors including a smaller sample size, shorter workshop, and shorter quiz length. Moreover, compared to neuro-ophthalmology, pediatric ophthalmology topics are infrequently covered in the preclinical curriculum at our institution. Therefore, it was unsurprising that the students on the pediatric vs. neurology rotation had lower mean pre-test scores and perceived a more difficult assessment. Aligned with this, most pediatric clerkship students (> 70%) correctly answered the neonatal conjunctivitis question, likely because this is a commonly tested U.S. Medical Licensing Exam topic. From this, our data impart insights in how 20/20 SIM can facilitate course objectives. For example, institutions may consider adding the 20/20 SIM neuro-ophthalmology tool to their neurology pathophysiology and/or clerkship curriculum to reinforce previously taught ophthalmology knowledge. Meanwhile, the pediatric ophthalmology tool may be optimized to introduce important but less emphasized topics in either a pediatric rotation or subspecialty elective. As the pediatric clerkship students reported increased comfort in ophthalmology following use of the tool, these data, irrespective of absolute knowledge gain, suggest how students can still obtain benefit.

This cross-sectional study has several limitations. This was a single-site study, and therefore these findings may not be generalizable to other institutions whose ophthalmology and UME curricula differ. Importantly, our evaluation of the tool was limited to respondents’ reaction and gain of knowledge [30]. Owing to the brief curricular time devoted to ophthalmology on these rotations, we could not evaluate the tool’s effectiveness in behavior change or external application of knowledge [30]. While we tried to administer a 6-month post-test in the neurology clerkship, we obtained a poor response rate to evaluate knowledge retention. We evaluated the use of 20/20 SIM in a large-group workshop format; therefore, it is possible that respondents’ satisfaction with the tool may have been influenced by the group facilitator. Future studies evaluating 20/20 SIM tool in self-directed formats are needed to assess independent knowledge acquisition and user engagement. Lastly, while we examined the 20/20 SIM tool with non-ophthalmic trainees from three different disciplines, we acknowledge that other specialties, including emergency medicine and family medicine, may also encounter ophthalmic presentations. Accordingly, we have since created an ophthalmic emergency “On Call” series and hope to introduce this new addition, along with the existing content on 20/20 SIM, to other relevant fields. Despite these limitations, we believe our study reveals important insights on how the novel tool can be implemented at other institutions seeking to expand their ophthalmology curriculum in UME and graduate medical education.

## Conclusions

In this study, we piloted the use of an online, case-based learning tool (20/20 SIM, www.2020sim.com) in a workshop format, first with residents, followed by medical students during core clinical clerkships to increase ophthalmology education and reinforce learning. All cohorts reported high engagement and satisfaction with the novel educational resource. Medical students reported trends towards increased comfort or knowledge in ophthalmology, demonstrating how 20/20 SIM may help address curricular gaps. Of note, these workshops have continued to be a component of the core curriculum for all cohorts. Important next steps will include how these sustained curricular additions affect knowledge retention and comfort levels longitudinally as well as trainee recruitment into ophthalmology. Uniquely, this study focused on the use of 20/20 SIM in workshop format within the core curriculum; however, this strategy represents only one of several ways in how the website can be used. To realize maximal benefit, this institution has drawn content from the 20/20 SIM website to teach pediatric residents in their didactic curriculum, preclinical students in a virtual ophthalmology elective, and third- and fourth-year medical students during an in-person ophthalmology elective. Given the free online nature of the website, we hope that 20/20 SIM provides opportunities for institutions to flexibly adapt the tool to suit their curricular needs.

## Data Availability

The datasets used and/or analyzed during the current study are available from the corresponding author on reasonable request.

## Abbreviations

AAN: American Academy of Neurology
ACGME: Accreditation Council for Graduate Medical Education
AUPO: Association of University Professors of Ophthalmology
AY: Academic year
CBL: Case-based learning
COMSEP: Council on Medical Student Education in Pediatrics
COVID-19: Coronavirus disease 2019
FOAMed: Free open access medical education
HPI: History of present illness
ICO: International Council of Ophthalmology
IM: Internal medicine
LCME: The Liaison Committee on Medical Education
PGY: Postgraduate year
REDCap: Research Electronic Data Capture
SD: Standard deviation
UME: Undergraduate medical education
U.S.: United States
